# Cervical Cancer Outcomes in Women With HIV in the Age of Antiretroviral Therapy

**DOI:** 10.1001/jamanetworkopen.2025.27389

**Published:** 2025-08-15

**Authors:** Alison K. Yoder, Rehema J. Thomas, Austin Huang, Anushka Mandalapu, Dana M. Roque, Kristina Bowles, Kevin Albuquerque, Christina Son, Michelle S. Ludwig, Kimberly Levinson, Anna E. Coghill, Elizabeth Yu Chiao, Lilie L. Lin

**Affiliations:** 1 Department of Radiation Oncology, The University of Texas MD Anderson Cancer Center, Houston; 2 Baylor College of Medicine, Houston, Texas; 3 Department of Gynecologic Oncology, University of Maryland, College Park; 4 Department of Cancer Epidemiology, University of Central Florida Moffitt Cancer Center, Tampa; 5 Department of Radiation Oncology, University of Texas Southwestern, Dallas; 6 Department of Radiation Oncology, University of Illinois–Chicago, Chicago; 7 Department of Radiation Oncology, Baylor College of Medicine, Houston, Texas; 8 Department of Gynecologic Oncology, Johns Hopkins University, Houston, Texas; 9 Department of Epidemiology, University of Texas MD Anderson Cancer Center, Houston; 10 Department of Radiation & Cellular Oncology, University of Chicago, Chicago, Illinois

## Abstract

This cross-sectional study investigates the outcomes for women living with HIV and cervical cancer in the US after the emergence of antiretroviral therapy.

## Introduction

Women living with HIV (WLWH) experience disproportionately high rates of cervical cancer.^1,2^ Whether outcomes and toxic effects are similar between WLWH and women living without HIV (WWH) in the age of highly active antiretroviral therapy (HAART) is unclear.^3,4,5,6^ Research on HIV and cervical cancer outcomes in the US is relatively scarce, with few studies examining disease progression or control. This study aims to fill this research gap.

## Methods

WLWH treated for cervical cancer from January 1997 through November 2017 were included in this cross-sectional study and retrospective multi-institutional review; WLWH were matched within each institution with WWH at a 1:3 ratio; a 1:2 ratio was used when a third matched patient was unavailable (eMethods in Supplement 1). The study was approved by the MD Anderson Cancer Center institutional review board. For WLWH, patients were included if they had a positive HIV test or known HIV infection at or before cancer diagnosis. Adherence to HAART for WLWH during treatment was determined via the medical record. If adherence could not be ascertained, those patients were deemed unknown and grouped with the nonadherent patients. Cox regression survival analysis was used to assess for potential associations between HIV status and adherence to ART -exposure categories: WLWH-adherent (WLWH-A), WLWH-nonadherent (WLWH-N), or WWH— clinical variables, and local control (LC), distant metastasis-free survival (DMFS), disease-free survival (DFS) and overall survival (OS). All statistical analyses were conducted using SPSS version 25.0. Statistical significance was assessed at p  < .05. This study was approved by each institution’s institutional review board and a waiver of consent was approved by each respective institution given the retrospective design in accordance with 45 CFR §46. The study followed STROBE reporting guidelines.

## Results

A total of 62 WLWH and 172 matched WWH with known cervical cancer, disease stage at diagnosis, and at least 1 follow-up clinical note were included (Table). Median follow-up time for the cohort was 47 months (IQR, 39-55 months). Most patients presented with stage III disease (120 patients [51%]), and (117 patients [50%]) underwent chemoradiation. Among the entire cohort, DFS and OS differed by HIV status and adherence to HAART (Figure), with worse survival for WLWH-N. More advanced disease stage and nonadherence to HAART were associated with worse OS in multivariable analysis. Similarly, among the subset of 86 patients who received definitive chemoradiotherapy, inferior OS and DFS were noted for WLWH-N. LC and DMFS were similar for both cohorts regardless of HIV status and adherence to HAART.

**Table.** Demographics and Treatment Characteristics for the Entire Cohort and for Patients Who Received Definitive Chemoradiation

Characteristic	Entire cohort				Definitive chemoradiation			
	No. (%)			*P* value	No. (%)			*P* value
	WWH (n = 172)	WLWH-A (n = 24)	WLWH-N (n = 38)		WWH (n = 68)	WLWH-A (n = 9)	WLWH-N (n = 9)	
No. of patients	172	24	38	NA	68	9	9	NA
Race and ethnicity								
Black	62 (36.0)	19 (79.2)	32 (84.2)	<.001^a^	25 (36.7)	8 (89.9)	5 (56.6)	.05^a^
Hispanic	39 (22.7)	3 (12.5)	3 (7.9)		29 (42.6)	1 (11.1)	3 (33.3)	
White and/or Asian^a^	71 (41.3)	2 (8.3)	3 (7.9)		14 (20.5)	0	1 (11.1)	
Smoking status								
Active	58 (33.7)	8 (33.3)	20 (52.6)	.04	18 (26.5)	1 (11.1)	3 (33.3)	.02^b^
Never	80 (46.5)	11 (45.8)	15 (39.5)		34 (50.0)	7 (78.8)	6 (66.7)	
Prior	34 (19.8)	4 (16.7)	2 (5.3)		16 (23.5)	0	0	
Unknown	0	1 (4.2)	1 (2.6)		0	1 (11.1)	0	
Disease stage								
I	58 (33.7)	9 (37.5)	9 (23.7)	.23^b^	1 (1.5)	0	0	.99^b^
II	22 (12.8)	5 (20.8)	2 (5.3)		16 (23.5)	2 (22.2)	2 (22.2)	
III	87 (50.6)	9 (37.5)	24 (63.2)		50 (73.5)	7 (78.8)	7 (77.7)	
IV	5 (2.9)	1 (4.2)	3 (7.9)		1 (1.5)	0	0	
Positive lymph nodes								
No	99 (57.6)	17 (70.8)	20 (52.6)	.35^b^	22 (32.3)	4 (44.4)	2 (22.2)	.60^b^
Yes	73 (42.4)	7 (29.2)	18 (46.4)		46 (68.7)	5 (56.6)	7 (77.7)	
Simplified histology								
Adenocarcinoma/other	30 (17.4)	2 (8.3)	6 (15.8)	.52^b^	6 (8.8)	0	1 (11.1)	.62^b^
SCC	142 (82.6)	22 (91.7)	32 (84.2)		62 (91.2)	9 (100.0)	8 (88.9)	
Chemotherapy used								
No	57 (33.1)	11 (45.8)	18 (47.4)	.16^b^	NA	NA	NA	NA
Yes	115 (66.9)	13 (54.2)	20 (52.6)		68 (100.0)	9 (100.0)	9 (100.0)	
Chemotherapy cycles, median (IQR)	5 (4-6)	4 (1-7)	3 (1-7)	.006^c^	5 (4-6)	4 (1-7)	4 (1-7)	.07^c^
Treatment era								
1997-2001	16 (9.3)	0 (0.0)	7 (18.4)	.11^b^	1 (1.5)	0	0	.23^b^
2000-2008	48 (27.9)	6 (25.0)	12 (31.6)		7 (10.3)	0	3 (33.3)	
2009-2017	108 (62.8)	18 (75.0)	19 (50.0)		60 (88.2)	9 (100.0)	6 (66.7)	
ART treatment compliance								
Yes	NA	24 (100)	NA	NA	NA	9 (100.0)	NA	NA
No	NA	NA	20 (52.6)	NA	NA	NA	6 (66.7)	NA
Unknown	NA	NA	18 (47.4)	NA	NA	NA	3 (33.3)	NA
Institution								
Baylor College of Medicine	30 (17.4)	5 (20.8)	4 (10.5)	.06^b^	26 (38.2)	4 (44.4)	3 (33.3)	.25^b^
Johns Hopkins	72 (41.9)	3 (12.5)	16 (42.1)		11 (16.2)	0	1 (11.1)	
University of Maryland	12 (7.0)	3 (12.5)	5 (13.2)		3 (4.4)	0	0	
MD Anderson	20 (11.6)	3 (12.5)	6 (15.8)		9 (13.2)	1 (11.1)	1 (11.1)	
Moffitt	17 (9.9)	6 (25.0)	2 (5.3)		4 (5.9)	3 (33.3)	0	
Southwestern	12 (7.0)	1 (4.2)	5 (13.2)		12 (17.6)	1 (11.1)	4 (44.4)	
University of Illinois-Chicago	9 (5.2)	3 (12.5)	0		3 (4.4)	0	0	
Treatment sequence								
Chemotherapy and RT	91 (52.9)	11 (45.8)	15 (39.5)	.14^b^	NA	NA	NA	NA
RT alone	13 (7.6)	2 (8.3)	10 (26.3)		NA	NA	NA	
Surgery alone	37 (21.5)	8 (33.3)	6 (15.8)		NA	NA	NA	
Surgery followed up RT	7 (4.1)	1 (4.2)	2 (5.3)		NA	NA	NA	
Surgery followed up Chemotherapy	2 (1.2)	0	0		NA	NA	NA	
Surgery followed up ChemoRT	22 (12.8)	2 (8.3)	5 (13.2)		NA	NA	NA	
RT dose, median (IQR)	NA	NA	NA	NA	45 (39.6-50.4)	45 (40.7-49.3)	45 (42.3-47.7)	.53^c^
RT fx, median (IQR)	NA	NA	NA	NA	25 (23-27)	25 (24-26)	25 (23-27)	.30^c^
Brachytherapy dose, median (IQR)	NA	NA	NA	NA	30 (17-43)	30 (17-43)	29 (18-40)	.95^c^
Brachytherapy fx, median (IQR)	NA	NA	NA	NA	2 (1-5)	2 (1-5)	3 (1-5)	.68^c^
Extended field RT	NA	NA	NA	NA	35 (52)	3 (33)	5 (63)	.46^b^
Radiation length, median (IQR), d	NA	NA	NA	NA	56 (40-72)	53 (34-72)	59 (49-69)	.12^c^
RT type								
3D-conformal	NA	NA	NA	NA	28 (41.2)	3 (33.3)	4 (44.4)	.88^b^
IMRT	NA	NA	NA		33 (48.5)	4 (44.4)	4 (44.4)	
Other or not specified	NA	NA	NA		7 (10.3)	2 (22.2)	1 (11.1)	
Brachytherapy type								
Interstitial needles	NA	NA	NA	NA	5 (7.3)	1 (11.1)	0	.50^b^
Other or not specified	NA	NA	NA		3 (4.4)	0	0	
Tandem and ring	NA	NA	NA		1 (1.5)	1 (11.1)	0	
Tandem and ovoid	NA	NA	NA		59 (86.8)	7 (77.8)	9 (100.0)	

Abbreviations: 3D, three dimensional; ART, antiretroviral therapy; fx, fractions; IMRT, intensity-modulated radiotherapy; NA, Not applicable; RT, radiotherapy; SCC, squamous cell carcinoma; WWH, Women without HIV; WLWH-A, Women living with HIV, HIV-positive adherent to antiretroviral therapy; WLWH-N, Women living with HIV, HIV-positive not adherent to highly active antiretroviral therapy.

^a^ This group was created as the Asian cohort was too small for statistical analysis. Thus, the decision was made to group this cohort with the White cohort of patients.

^b^ Indicates χ^2^ test.

^c^ Indicates nonparametric test.

**Figure.**
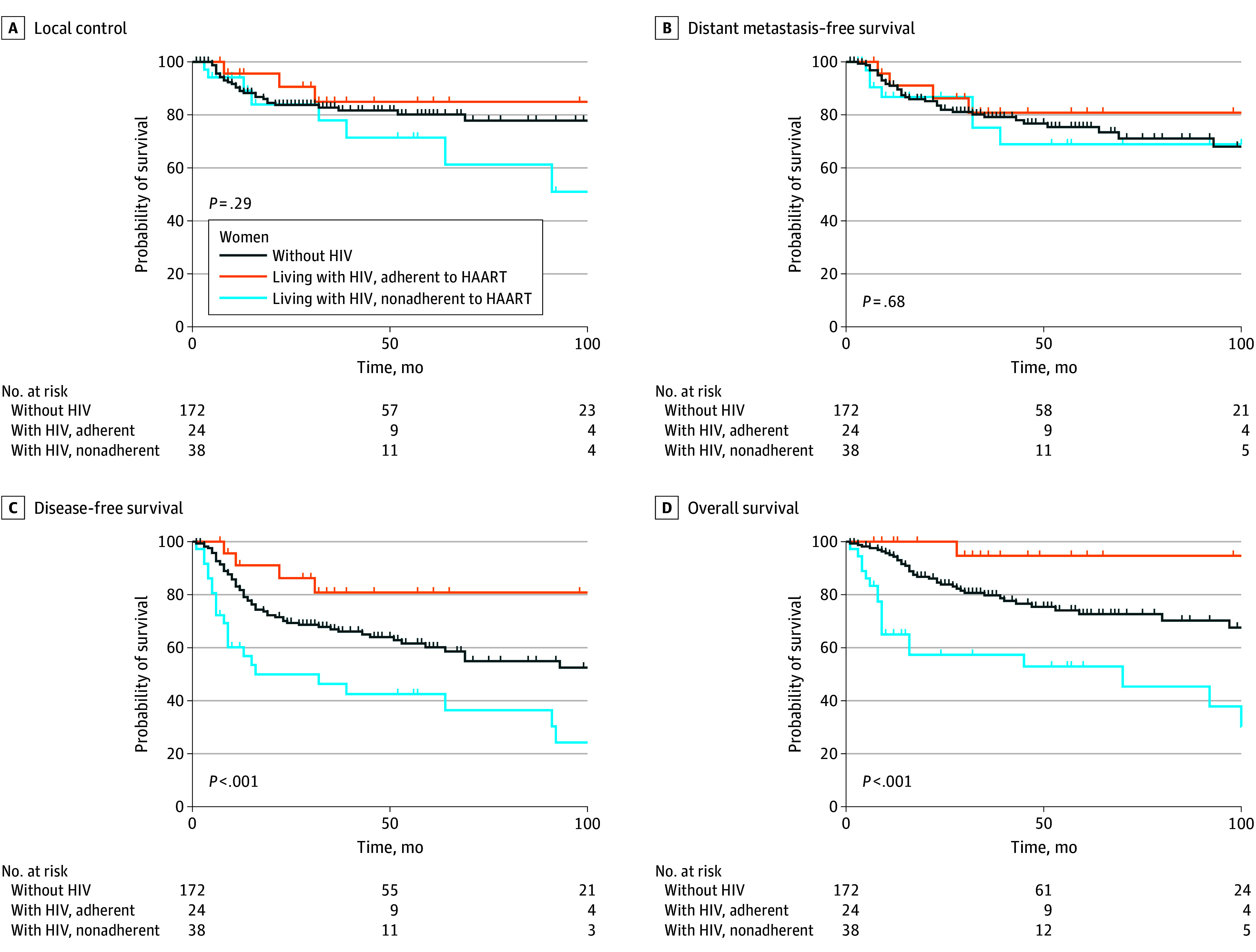
Kaplan-Meier Curves Comparing Local Control, Distant Metastasis-Free Survival, Disease-Free Survival, and Overall Survival for Women Without HIV (WWH), Women Living With HIV Who Adhered to Antiretroviral Therapy (WLWH-A), and WLWH Who Were Nonadherent to ART (WLWH-N) Local recurrence was defined as disease recurrence within the cervix, vagina, parametria, or regional nodes. Distant recurrence was defined as disease recurrence in another organ or nonregional lymph node. Disease-free survival was defined from the date of initial biopsy confirming cervical cancer to the date of first recurrence or last follow-up. Five-year local control rates did not differ by HIV status and adherence to ART (WWH, 80% [95% CI, 72%-86%], WLWH-A, 84% [95% CI, 60%-94%], and WLWH-N, 72% [95% CI, 45%-87%]; *P* = .29), and neither did DMFS (WWOH, 76% [95% CI, 67%-82%], WLWH-A, 81% [95% CI, 56%-92%], and WLWH-N, 69% [95% CI, 44%-85%]; *P* = .68). Five-year DFS was 60% (95% CI, 51%-68%) in WWH, 81% (95% CI, 56%-92%) in WLWH-A patients, and 43% (95% CI, 25%-59%) in WLWH-N patients (*P* < .001). Five-year OS was 73% (95% CI, 63%-80%) in WWH, 95% (95% CI, 69%-99%) in WLWH-A, and 53% (95% CI, 34%-69%) WLWH-N patients, respectively (*P* < .001). Nonadherence with ART was associated with worse DFS (WLWH-A HR, 0.41 [95% CI, 0.15-1.12]; *P* = .08; WLWH-N HR, 1.81 [95% CI, 1.10-2.98]; *P* = .02), and OS (WLWH-A HR, 0.16 [95% CI, 0.02-1.18]; *P* = .07; WLWH-N HR, 2.34 [95% CI, 1.32-4.15]; *P* = .004) on multivariate analysis.

The frequency of acute grade 3 or higher gastrointestinal or genitourinary toxic effects did not differ for WWH (n = 14,14%), WLWH-A (n = 2,13%), and WLWH-N (n = 4,19%) patients (*P* = .82). Similarly, among the patients who received definitive chemoradiotherapy, no significant difference was found in the frequency of acute grade 3 or higher gastrointestinal or genitourinary toxic effects between WWH (n = 11, 21%), WLWH-A v, and WLWH-N (n = 2, 40%) patients (*P* = .26).

## Discussion

To our knowledge, this cross-sectional study is the largest to date evaluating the association of HIV infection with cervical cancer outcomes in the US. In this study, we found no statistically significant differences in outcomes between WLWH who adhered to HAART and WWH. However, WLWH-N had significantly worse DFS and OS than WLWH-A and WWH in both the entire cohort as well as in the group treated with definitive chemoradiation. No differences in gastrointestinal or genitourinary toxic effects were evident between these 2 cohorts, including in the subset of women who received chemoradiotherapy.

Limitations include the retrospective design, extension over several treatment eras, and limited patient numbers (particularly in the chemoradiation subset). Despite these limitations, this study provides valuable insights into the importance of HAART adherence during treatment for cervical cancer, which should be investigated in future studies. This is the largest cohort of WLWH treated for cervical cancer in the US and the inclusion of 7 institutions with different practice patterns also helps to reduce possible treatment bias.

In the era of HAART, WLWH diagnosed with cervical cancer should receive curative treatment per standard guidelines. The importance of adhering to HAART during treatment for cervical cancer should be stressed by oncologists to ensure that WLWH have optimal outcomes.

## Notes

Supplement 1.
eMethods

Supplement 2.
Data Sharing Statement

## References

[1] Kuhn L, Wang C, Tsai WY, Wright TC, Denny L. Efficacy of human papillomavirus-based screen-and-treat for cervical cancer prevention among HIV-infected women. AIDS. 2010;24(16):2553-2561. doi:10.1097/QAD.0b013e32833e163e 20706107

[2] Abraham AG, D’Souza G, Jing Y, ; North American AIDS Cohort Collaboration on Research and Design of IeDEA. Invasive cervical cancer risk among HIV-infected women: a North American multicohort collaboration prospective study. J Acquir Immune Defic Syndr. 2013;62(4):405-413. doi:10.1097/QAI.0b013e31828177d7 23254153 PMC3633634

[3] Ferreira MP, Coghill AE, Chaves CB, . Outcomes of cervical cancer among HIV-infected and HIV-uninfected women treated at the Brazilian National Institute of Cancer. AIDS. 2017;31(4):523-531. doi:10.1097/QAD.0000000000001367 28060014 PMC5263104

[4] Meghani K, Puri P, Bazzett-Matabele L, . Significance of HIV status in cervical cancer patients receiving curative chemoradiation therapy, definitive radiation alone, or palliative radiation in Botswana. Cancer. 2024;130(14):2462-2471. doi:10.1002/cncr.35289 38529676

[5] Dryden-Peterson S, Bvochora-Nsingo M, Suneja G, . HIV infection and survival among women with cervical cancer. J Clin Oncol. 2016;34(31):3749-3757. doi:10.1200/JCO.2016.67.9613 27573661 PMC5477924

[6] Simonds HM, Wright JD, du Toit N, Neugut AI, Jacobson JS. Completion of and early response to chemoradiation among human immunodeficiency virus (HIV)-positive and HIV-negative patients with locally advanced cervical carcinoma in South Africa. Cancer. 2012;118(11):2971-2979. doi:10.1002/cncr.26639 22072021 PMC3448067

