# Supplementary material for: Cervical Cancer Outcomes in Women With HIV in the Age of Antiretroviral Therapy
Source: JAMA Netw Open. 2025 Aug 15;8(8):e2527389. doi: 10.1001/jamanetworkopen.2025.27389 (PMC12357181; doi:10.1001/jamanetworkopen.2025.27389)
Supplement: Supplement 1. — eMethods [file jamanetwopen-e2527389-s001.pdf]

Supplemental Online Content

Yoder AK, Thomas RJ, Huang A, et al. Cervical cancer outcomes in women with HIV in the age of antiretroviral therapy. *JAMA Netw Open.* 2025;8(8):e2527389. doi:10.1001/jamanetworkopen.2025.27389

Supplement 1. eMethods

This supplemental material has been provided by the authors to give readers additional information about their work.

© 2025 Yoder AK et al. *JAMA Network Open*.

eMethods:

After approval by the institutional review boards at each institution, we undertook a retrospective review adhering to STROBE guidelines of WLWH who were treated at seven U.S.-based cancer centers for cervix cancer from 1/1997 – 11/2017: MD Anderson Cancer Center, Johns Hopkins, Baylor College of Medicine, University of Maryland, Moffitt Cancer Center, University of Texas Southwestern, and University of Illinois-Chicago. A waiver of consent was approved by the IRB from each institution given the retrospective design. Data was analyzed in 2023-2024. We included adults aged $\geq$ 18 with histologically confirmed cervix cancer and known disease stage at diagnosis who had been treated with surgical resection or RT. For WLWH, patients were included if they had a positive HIV infection documented in the electronic medical record. The positive test must have occurred before or at the time of cancer diagnosis, prior to initiation of therapy. Patients with an HIV diagnosis more than 1 month after cancer diagnosis were excluded.

Treatment era was defined based on class of ART available as follows: 1997-2001, when ART became widely available and non-nucleoside antiretroviral therapies were the most common; 2002-2008, when integrase inhibitors became widely utilized; and 2009-2017, or the modern age of ART (12).

Demographic variables and HIV-related variables (if applicable) were abstracted from the electronic medical record for each patient by the research staff. Race and ethnicity were determined via electronic health record. Patients of Hispanic ethnicity were grouped together. This was assessed as a covariate as previous studies have shown disparate outcomes between racial and ethnic groups. Treatment toxicities and disease outcomes were extracted as well. Toxicities were scored based on the Common Terminology Criteria for Adverse Events v4.03. Follow-up time for survival analyses was computed from the date of the initial biopsy until date

© 2025 Yoder AK et al. *JAMA Network Open*.

of the outcome or last follow up. Disease recurrence data was based on the date of biopsy, if available. If biopsy was unavailable, then date of positive imaging or physical exam was used as the date of recurrence. Local recurrence was defined as disease recurrence within the cervix, vagina, parametria, or regional nodes. Distant recurrence was defined as disease recurrence in another organ or non-regional lymph node.

Demographics, treatment variables, and toxicities were compared between WLWH who adhered to ART (WLWH-A), WLWH who did not adhere to ART (WLWH-N), and WwoH by using Chi-square tests or independent *t*-tests. Kaplan-Meier analysis was used to compare local-recurrence free survival (LRFS), distant-metastasis-free survival (DMFS), disease-free survival (DFS), and overall survival (OS) between cohorts. The log-rank test was used to test for difference between curves. Cox regression survival analysis was used to assess for potential associations between HIV status and adherence to ART (exposure categories: WLWH-A, WLWH-N, or WwoH), clinical variables, and LRFS, DMFS, DFS and OS. Backwards step-wise multivariable analyses were done; variables were included in the initial Cox model if $P<0.20$; and variables were kept in the final model assessing the impact of HIV if $P<0.05$.

A subset analysis was then completed assessing women who received definitive-intent chemoradiation for their cervical cancer. Definitive intent was defined as an external-beam radiation dose of at least 40 Gray (Gy), at least one brachytherapy procedure, and at least one cycle of chemotherapy. All statistical analyses were completed in IBM SPSS version 26.0. Statistical significance was defined as $P<0.05$.

© 2025 Yoder AK et al. *JAMA Network Open*.
