# Supplementary material for: Cervical Cancer Outcomes in Women With HIV in the Age of Antiretroviral Therapy
Source: JAMA Netw Open. 2025 Aug 15;8(8):e2527389. doi: 10.1001/jamanetworkopen.2025.27389 (PMC12357181; doi:10.1001/jamanetworkopen.2025.27389)
Supplement: Supplement 2. — Data Sharing Statement [file jamanetwopen-e2527389-s002.pdf]

Data Sharing Statement

Yoder. Cervical Cancer Outcomes in Women With HIV in the Age of Antiretroviral Therapy. *JAMA Netw Open*. Published August 15, 2025. doi:10.1001/jamanetworkopen.2025.27389

Data
Data available: Yes
Data types: Deidentified participant data
How to access data: De-identified data will be available will be availablef or researchers whose proposed use of the data has been approved by emailing Lilie Lin at lllin@mdanderson.org
When available: With publication

Supporting Documents
Document types: None

Additional Information
Who can access the data: researchers whose proposed use of the data has been approved
Types of analyses: for an approved research purpose
Mechanisms of data availability: after approval of a proposal, with a signed data access agreement with investigator support
